# Revealing genetic drivers of ovarian cancer and chemoresistance: insights from whole-genome CRISPR-knockout library screens

**DOI:** 10.1007/s13402-025-01102-4

**Published:** 2025-08-28

**Authors:** Tali S. Skipper, Kristie-Ann Dickson, Christopher E. Denes, Matthew A. Waller, Tian Y. Du, G. Gregory Neely, Nikola A. Bowden, Alen Faiz, Deborah J. Marsh

**Affiliations:** 1 Translational Oncology Group, School of Life Sciences, Faculty of Science, University of Technology Sydney, Ultimo, NSW Australia; 2 Dr. John and Anne Chong Lab for Functional Genomics, Charles Perkins Centre, School of Life and Environmental Sciences, University of Sydney, Camperdown, NSW Australia; 3 School of Medicine and Public Health, University of Newcastle, Newcastle, NSW Australia; 4 Respiratory Bioinformatics and Molecular Biology Group, School of Life Sciences, Faculty of Science, University of Technology Sydney, Ultimo, NSW Australia

**Keywords:** Epithelial ovarian cancer, CRISPR-knockout library screen, Precision medicine, Synthetic lethality, Drug response

## Abstract

Understanding genetic dependencies in cancer is key to identifying novel actionable drug targets to advance precision medicine. Whole-genome CRISPR-knockout library screening methods have facilitated this goal. Pooled libraries of single guide RNAs (sgRNAs) targeting over 90% of the annotated protein coding genome are used to induce gene knockouts in pre-clinical cancer models. Novel genes of interest are identified by evaluating sgRNA dropout or enrichment following selection pressure application. This method is particularly beneficial for researching cancers where effective treatment strategies are limited. One example of a commonly chemoresistant cancer, particularly at relapse, is the low survival malignancy epithelial ovarian cancer (EOC), made up of multiple histotypes with distinct molecular profiles. CRISPR-knockout library screens in pre-clinical EOC models have demonstrated the ability to predict biomarkers of treatment response, identify targets synergistic with standard-of-care chemotherapy, and determine novel actionable targets which are synthetic lethal with cancer-associated mutations. Robust experimental design of CRISPR-knockout library screens, including the selection of strong pre-clinical cell line models, allows for meaningful conclusions to be made. We discuss essential design criteria for the use of CRISPR-knockout library screens to discover genetic dependencies in cancer and draw attention to discoveries with translational potential for EOC.

**Supplementary Information:**

The online version contains supplementary material available at 10.1007/s13402-025-01102-4.

## Clinical trial number

Not applicable.

## Introduction

Dissecting the genetic landscape of cancers is crucial for the advancement of precision medicine, a goal that has been facilitated by the advent of CRISPR-Cas9 (Clustered Regularly Interspaced Short Palindromic Repeat) gene editing [1]. Here, a single guide RNA (sgRNA) localises a Cas9 endonuclease to a targeted DNA sequence causing a double strand-break (DSB). DSB repair through endogenous, error prone DNA repair pathways often result in base insertion and/or deletion, likely causing a frame-shift mutation leading to gene knockout (KO). Consequently, phenotypic effects associated with single gene perturbations can be evaluated.

Human pooled whole-genome CRISPR-Cas9 KO library screens (herein referred to as CRISPR-KO library screens) are a high-throughput adaptation of single gene CRISPR-Cas9 editing [2, 3]. A pooled library of sgRNAs targeted to over 90% of the protein coding genome are used to generate a mixed KO population. By comparing sgRNA abundance between differently selected cell populations, a list of candidate genes whose KO induces a significant phenotypic alteration can be generated. CRISPR-KO libraries can therefore facilitate a precision medicine approach to identifying genetic dependencies that can be exploited for the treatment of cancer, enabling particular value in those with limited effective therapeutic options such as epithelial ovarian cancer (EOC).

EOC makes up over 90% of all ovarian cancers and is further classified into 5 major, genetically distinct histotypes: high-grade serous ovarian cancer (HGSOC; up to 75%), endometrioid ovarian cancer (EnOC; ~10%), ovarian clear cell carcinoma (OCCC; ~6%), low-grade serous ovarian cancer (LGSOC; <5%), and mucinous ovarian cancer (MOC; <5%) [4]. Frequencies of some differing between countries [5]. Despite distinct molecular profiles, the current standard-of-care for all EOCs involves surgical debulking and combinations of platinum-based chemotherapies, including carboplatin and the microtubule stabilizing agent paclitaxel [6].

Although EOC can be treated successfully if diagnosed at Stage I (pre-metastatic disease), the non-descript symptomatic presentation and lack of early detection methods often results in detection at advanced stages (Stages III and IV) following metastasis [7]. Globally, ovarian cancer accounts for 31% of deaths from gynaecological cancer despite only causing 22% of cases, highlighting ovarian cancer as a highly lethal gynaecological malignancy [8]. Consequently, exploring novel therapeutic avenues is essential for improving patient prognosis and quality of life.

The use of poly (ADP-ribose) polymerase (PARP) inhibitors for the treatment of advanced EOC with homologous-recombination (HR) deficiencies has been the most impactful advancement in EOC treatment in recent years and has provided considerable evidence supporting the value of precision medicine [9]. PARP inhibition of HR deficient cancer cells, for example, those with *BRCA1* or *BRCA2* mutations or a “BRCAness” phenotype as a result of mutations in *RAD51C*,* RAD51D*,* ATM*,* PALB2*, and other genes encoding members of the HR pathway, results in synthetic lethality of cancer cells, whereby the combined effect of treatment and mutation results in cell death [10]. These mutations are seen in approximately 50% of HGSOC [10], although the evaluation of a patients’ “BRCAness” based on alternative HR-deficiency genetic mutations, could expand the use of PARP inhibitors to other histotypes [11]. Other advances in precision medicine approaches to treating EOC include use of the mitogen-activated protein kinase kinase (MEK) inhibitor trametinib for treatment of LGSOC harbouring *KRAS* and *BRAF* mutations [12]. In 2023, trametinib was approved by the National Health Service England for the treatment of recurrent, progressive LGSOC, with the potential for global approval [13, 14].

CRISPR-KO library screens have been used in a number of pre-clinical cancer models for early-stage target discovery, acting as a valuable tool in the drug development pipeline [15–17]. At present, many CRISPR-KO library screens with an EOC focus lack clear distinction of histological subtype leaving a gap in the literature for researchers to screen alternative pre-clinical models. Here we review the use of whole-genome CRISPR-KO library screens in pre-clinical models of ovarian cancer thus far, outlining key technical considerations of this research, and emphasising the need for research to be conducted in models representative of defined histological subtypes. Further, we highlight the most promising results with high translational potential discovered using this technique so far.

## Workflow for human, pooled whole-genome CRISPR-KO library screens

**Fig. 1:**
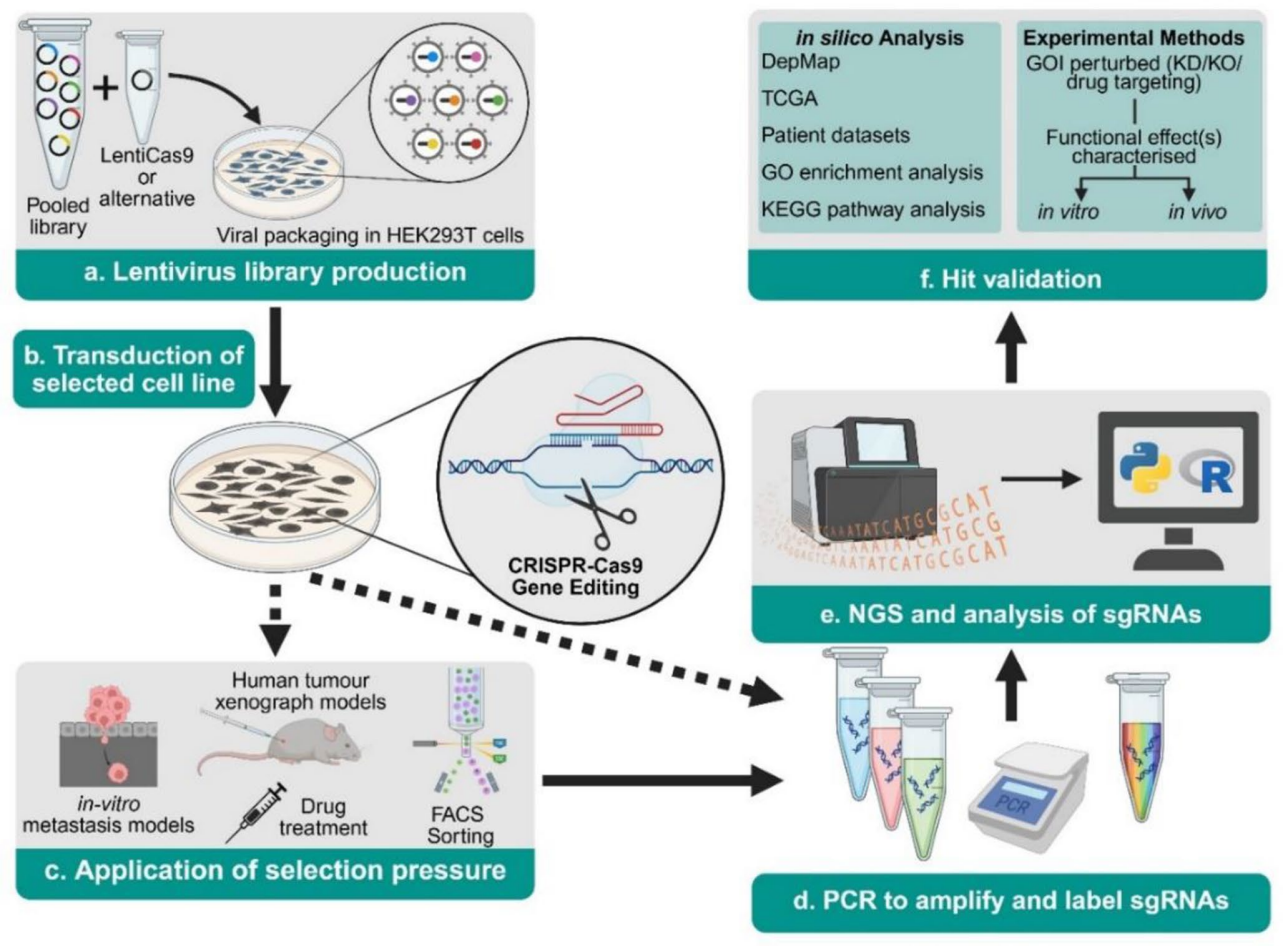
Pooled CRISPR-knockout library workflow. (**a**) sgRNA libraries are packaged into a viral vector delivery system and (**b**) transduced into a cell line of interest. (**c**) Selection pressure designed to interrogate a specific phenotype is applied to transduced cells. (**d**) Genomic DNA is extracted and sgRNA sequences incorporated into the genome are amplified, barcoded, and pooled for next generation sequencing (NGS). (**e**) NGS data is analysed to identify sgRNAs which have been enriched or depleted following application of selection pressure, and (**f**) “hits” are validated using a range of in silico, in vitro, and in vivo techniques. Abbreviations: DepMap (Cancer Dependency Map), FACS (Fluorescence Activated Cell Sorting), GO (Gene Ontology), GOI (Gene of Interest), KD (knockdown), KEGG (Kyoto Encyclopedia of Genes and Genomes), KO (knockout), PCR (Polymerase Chain Reaction), sgRNA (single guide RNA), TCGA (The Cancer Genome Atlas)

Pooled whole-genome CRISPR-KO library screens follow a standard workflow (Fig. 1). sgRNA libraries encoded in plasmids are packaged into a lentiviral delivery system for transduction into a cell model of interest [18]. Transduction occurs at a low multiplicity of infection (MOI), with 0.3 to 0.5 most commonly used in the EOC research discussed [19]. This increases the likelihood of a single viral integration per cell and mitigates the chance of inadvertently screening for multi-gene interactions [2]. Transduced cells undergo antibiotic selection and are maintained in culture to allow for the effects of CRISPR-Cas9 gene editing to become apparent. Following transduction, a selection pressure can be applied to interrogate KOs responsible for a phenotype of interest, details of which are discussed in the following sections. Genomic DNA is extracted from transduced cells and sgRNA sequences are amplified, barcoded, and pooled for next-generation sequencing (NGS). By comparing sgRNA abundance (enrichment or depletion) between those cells exposed to a selection pressure and control samples, genes of interest (GOIs) are identified. In depth protocols relating to this workflow have been previously published [18, 20–26].

The CRISPR-KO library screen workflow has a broad range of applications dependent on the model system in which it is used. Whilst this review aims to contextualise the use of CRISPR-KO library screens within the context of EOC (Table 1), the discussed methods can be used as a framework for studies in other cancers, and particularly as a starting point for those malignancies with limited therapeutic opportunities.

**Table 1:** Whole-genome CRISPR-KO library design in published EOC screens

Histotype	Cell Line(s)	sgRNA Library	NGS data analysis method	Study aims	CRISPR-KO library prioritised GOIs	Refs.
HGSOC	OVCAR-8	Brunello	MAGeCK and DESeq2	Identify targets for sensitising cells to carboplatin	*CSNK2A2*	[27]
HGSOC	OVCAR-8	Brunello	Bowtie, Fisher Exact test, CRISPRscreen	Identify targets for reducing CD24 expression	*GPAA1*	[28]
HGSOC	Kuramochi, OVSAHO	Avana4	STARS	Identify standard-of care resistance mechanisms	*BCL2L1*	[29]
HGSOC	OVCAR-3	Brunello	MAGeCK	Identify cancer response mechanisms to hydroxychloroquine (HCQ)	N/A	[30]
HGSOC	PEO1	GeCKOv2	DESeq2 and MAGeCK	Identify genes involved in anoikis resistance	*ACADVL* *ECHDC2*	[31]
HGSOC w/*BRAC1* mutations	UMB1.289, COV362, JHOS-2	GeCKOv2	STARS	Identify modifiers of cisplatin and olaparib sensitivity	*DYNLL1* *ATMIN*	[32]
LGSOC	VOA-6406, VOA-4627	Brunello	MAGeCK	Identify pathways involved in MEK inhibitor resistance	*MAP3K1* *SHOC2*	[33]
OCCC	JHOC-5, OSE3 (normal ovarian surface epithelial cell)	GeCKOv2	MAGeCK	Identify targets of OCCC proliferation	*DHX38*	[34]
OCCC	OVISE, ES-2**, TOV-21G, JHOC-5	TKOv3	MAGeCK	Identify targets of OCCC proliferation	*XPR1*	[35]
OCCC	OVISE, ES-2**, TOV-21G, JHOC-5	TKOv3	MAGeCK	Identify ARID1A/PIK3CA synthetic lethal targets	*KDM2A* *PAIP1*	[36]
OCCC	TOV-21G	Human Improved Genome-Wide Knockout CRISPR Library v1	MAGeCK	Identify biomarkers for ATR sensitivity in ARID1A mutated OCCC	PP2A subunits	[37]
OCCC	RMG-I isogenic pair	TKOv3	Bowtie 2, BAGEL2	Identify ARID1A synthetic lethal targets	*KEAP1*	[38]
HGSOC	A2780*	GeCKOv2	MAGeCK	Identify modifiers of olaparib sensitivity	*C12orf5* (TIGAR)	[39]
Undefined	A2780* SK-OV-3**	GeCKOv2	Not described	Identify cisplatin resistance genes	*ZNF587B* *SULF1*	[40]
Undefined	SK-OV-3**	GeCKOv2	RIGER, RSA	Identify druggable oncogenes	*KPNβ1*	[41]
Undefined	SK-OV-3**	GeCKOv2	MAGeCK	Identify prognostic markers of metastasis	*VSTM2L*	[42]
Undefined	SK-OV-3**	GeCKOv2	MAGeCK	Identify genes involved in elaiophylin-induced paraptosis	*PTPN11*	[43]
Undefined	SK-OV-3**	GeCKOv2	RIGER	Identify genes involved in peritoneal metastasis	*IL20RA*	[44]
Undefined	SK-OV-3**	GeCKOv2	Not described	Identify genes involved in metastasis	*FCGR1A*	[45]
Undefined	SK-OV-3**	GeCKOv2	MAGeCK	Identify genes regulating ovarian cancer progression and peritoneal dissemination	*HTR1E*	[46]
Undefined	SK-OV-3**	GeCKOv2	RIGER	Identify genes involved in peritoneal metastasis	*ITK*	[47]
Undefined	SK-OV-3**	GeCKOv2	RIGER	Identify genes involved in anoikis resistance	*PCMT1*	[48]
Undefined	SK-OV-3**	GeCKOv2	MAGeCK	Identify regulators of B7-H3	*SUPT20H*	[49]

The histotype of interest investigated in the publication is defined in column 1 with ‘undefined’ highlighting studies without a histotype focus. *Reclassified histotype. **Original histotype in query. N/A implies screen hits were not prioritised using CRISPR-KO library screening for further biological validation. Publications dating up until and including 12 February 2025

Abbreviations: BAGEL (Bayesian Analysis of Gene Essentiality), GeCKOv2 (Genome wide CRISPR-Cas9 Knock Out version 2), GOI (Gene of Interest), MAGeCK (Model-based Analysis of Genome-wide CRISPR-Cas9-Knockout), NGS (Next-generation sequencing), OCCC (Ovarian Clear Cell Carcinoma), RIGER (RNAi Gene Enrichment Ranking), RSA (Redundant siRNA activity), TKOv3 (Toronto Knock Out version 3)

## Construction and delivery of whole-genome sgRNA libraries

CRISPR-KO libraries are constructed of several independent sgRNAs per gene (3–10) to account for variations in sgRNA activity, off-target effects and/or chance integrations not associated with the evaluated phenotype [2, 23, 50]. This redundancy reduces false-negative potential and increases statistical power. As a result, whole-genome libraries are usually constructed of over 70,000 different sgRNAs carefully selected to maintain effective library coverage and improve overall hit accuracy [51]. A key factor for sgRNA design is ensuring that guides are targeted to genomic regions away from single nucleotide polymorphism (SNP) hotspots to ensure accurate gene targeting and obtain accurate representation following NGS. Several algorithms which consider genomic location, GC content, secondary structure, and nucleotide identity at both proximal and distal regions to the protospacer adjacent motif (PAM), exist to aid researchers in the design process [52, 53].

Along with specific gene targeting sgRNAs, sgRNA libraries usually contain negative control sgRNAs, essential for benchmarking screen performance and assessing background noise [23, 54]. The ability to evaluate neutral variations not attributed to phenotypic effect provides a platform for normalising NGS data and enables more accurate interpretations of gene-specific effects. Usually, negative control sgRNAs are non-targeting and constructed of random genomic sequences shown not to complement any area of the genome; however, poor annotation and high variability among some genomic regions can cause unintentional sequence-homology between random sgRNAs and endogenous sequences [55]. Instead, sgRNAs designed to target non-human reporter genes (e.g. EGFP, LacZ, and luciferase, as are included in the Toronto Knockout version 3 (TKOv3) library), may have a reduced potential for off-target effects as these sequences are not present within the human genome [56]. Alternatively, using intergenic control sgRNAs, designed to not affect protein-coding regions, adds a control for non-specific DNA-damage responses and can mitigate signalling related artifacts [54]. The libraries used in the discussed studies (Tables 1 and 2) do not contain intergenic control sgRNAs.

Whilst researchers can independently design human whole-genome sgRNA libraries, many opt to use one of over 20 sgRNA optimised and validated libraries deposited in AddGene [20]. At present, published whole-genome EOC studies have used the libraries outlined in Table 2. Detailed information regarding the design strategies of these libraries is provided in the articles referenced in Table 2. Despite minimal sequence identity between these libraries, differences in ability to detect known essential genes are negligible [50, 53]. Instead, the choice of CRISPR-KO library usually depends on coverage limitations, impacting on number of guides, and mode of Cas9/sgRNA delivery into the model of interest.

**Table 2:** Human whole-genome CRISPR-KO libraries used to date in studies of EOC

Library Name	No. Target Genes	No. sgRNAs (sgRNAs per gene)	Controls	Recommended lentiviral vector backbone(s)	Refs.
GeCKOv2	19,050	123,411 (6)	1,000 non-targeting sgRNAs	lentiCRISPR v2 (Cas9 expressing); lentiGuide-Puro	[57]
Avana4*	18,675	74,700 (4)	1,000 non-targeting sgRNAs	lentiCRISPR v2 (Cas9 expressing); lentiGuide-Puro	[51]
Brunello	19,114	76,441 (4)	1,000 non-targeting sgRNAs	lentiCRISPR v2 (Cas9 expressing); lentiGuide-Puro	[51]
Human Improved Genome-Wide Knockout CRISPR Library v1	18,010	90,709 (5)	No additional controls	pKLV2-U6gRNA5(BbsI)-PGKpuro2ABFP-W	[58]
TKOv3	18,053	70,948 (4)	142 control sgRNAs targeting EGFP, LacZ, and luciferase	lentiCRISPR v2 (Cas9 expressing); pLCKO2	[56]

Libraries are listed in chronological order aligned to their original publication. The libraries included are those used in studies up until and including 12th February 2025. All libraries are available through https://www.addgene.org with the exception of Avana4 (*)

Abbreviations: CRISPR (Clustered Regularly Interspaced Short Palindromic Repeat), GeCKOv2 (genome-scale CRISPR-KO version 2), sgRNA (single guide RNA), TKOv3 (Toronto KO version 3)

### sgRNA coverage

sgRNA coverage is defined as the minimum number of cells per sgRNA maintained throughout a CRISPR-KO library screen in order to achieve statistically significant results and is partly dictated by screen directionality [19]. Drop-out, or negative-selection, screens focus on the loss of sgRNAs between selected and unselected populations. Here, coverage is recommended to be greater than 500x (often as high as 1000x) to reduce signal-to-noise ratio, avoid genetic bottlenecks due to chance depletions, and avoid the loss of rare signals [25, 59]. Increasing coverage beyond 1000x is usually not recommended due to experimental feasibility and cost, and it is unlikely to benefit in information gain. Enrichment, or positive-selection, screens focus on the increased ratio of sgRNAs between selected and unselected populations. As most cells are expected to be negatively selected against, statistically significant enrichments can be detected at a lower coverage of 100-200x [19].

High coverage combined with multiple sgRNAs per gene means a large number of cells must be maintained throughout a screen. This can be challenging in models where cell number capabilities may be limited, such as cell lines with a slow growth rate or large size, screens with in vivo elements such as human xenograft models, or when using primary cells [25]. Consequently, screening parameters may need to be compromised to accommodate the selected model. Publicly available bioinformatic tools such as CRISPulator can be used to simulate conditions and inform on minimum recommended coverage based on individual screen parameters [59].

Alternatively, cell number limitations can be overcome by using a library with fewer sgRNAs per gene. Genome-scale CRISPR-KO version 2 (GeCKOv2) is supplied as two half libraries with 3 sgRNAs per gene in each, allowing a partial library to be used for this purpose [2, 41]. More recently, alternative CRISPR-KO libraries have been designed specifically with a reduced number of sgRNAs per gene. These include Humagne Set C and Set D Human CRISPR Knockout Libraries [60], Garnett Lab MinLibCas9 Library [61], and the Human Genome-Wide Reduced Double-gRNA Library [62]; all of which are available in the Addgene catalogue.

### Method of vector delivery

CRISPR-KO library screens require both Cas9 and the sgRNA library to be expressed in a model of interest. This can be achieved either by using a model engineered to stably express Cas9 and subsequent sgRNA library delivery, or by using an sgRNA vector backbone which also encodes Cas9. Whilst both methods have demonstrated efficient gene KO, library performance has been suggested to correlate with Cas9 expression which could be improved by separate Cas9 and sgRNA expression [19, 57, 63]. Nonetheless, concurrent expression of Cas9 and sgRNA may be more suited for in vivo screens or screens using primary cells. Commercial libraries are usually made to be compatible with a particular vector, details of which is supplied as part of technical documents, with those used in EOC studies referenced in Table 2 [20].

The ability of lentiviruses to integrate into dividing and non-dividing cells, create stable KOs for ease of generating NGS data, and deliver large and multi-gene cassettes has made them the ideal delivery method for CRISPR-KO library screens [64]; however, transduction efficiency is highly variable between cell lines and needs to be experimentally determined [65]. Furthermore, lentiviral transduction of primary cells often requires high titres associated with cytotoxicity and less consistent efficiency, and is yet to be extensively evaluated in primary ovarian cancer cells [66, 67]. Consequently, as novel pre-clinical EOC models are used in CRISPR-KO library screens, alternative delivery systems including electroporation and nanoparticle techniques, may need to be explored for genome-wide screening [64, 68].

## Suitability of pre-clinical cell line models for CRISPR-KO library screens

Cell line selection for CRISPR-KO library screens depends on the experimental design, methods for which are discussed in the following section. For example, many cell lines are unable to grow in vivo as tumour xenograft models, eliminating their use in screens with an in vivo design element [69]. Additionally, consideration of innate or engineered drug resistance is important for all screens where the aim is unmasking novel therapeutic avenues [70]. Interestingly, commercially available drug-resistant EOC cell line pairs, for example the EnOC cell lines A2780/A2780cisR [71], have not yet been used in parallel whole-genome CRISPR-KO library screens, leaving an opportunity for future use in investigations aimed at predicting and/or overcoming platinum drug resistance in a personalised medicine approach.

Furthermore, copy number alterations, seen in the majority of EOC cell line models, have been shown to have an effect on KO efficiency, with stronger loss-of-function enrichments seen in diploid over tetraploid cells and further preference given to haploid cell lines [72–74]. Whilst EOC cell lines cannot be selected based on optimal ploidy, acknowledging these alterations is key when interpreting CRISPR-KO library results in ovarian cancer cell line models. Information on the cell lines used in CRISPR-KO library screens to study ovarian cancer to date is summarised in Online Resource 1, with additional information documented in Cell Model Passports which functions as a data hub for the Cancer Dependency Map at the Wellcome Sanger Institute [75].

### Molecular disparity in pre-clinical EOC models

Impactful conclusions from CRISPR-KO library screens are drawn from screens using models which accurately reflect the disease in question. This is especially important within the context of EOC, given that there are multiple histological subtypes defined by characteristic molecular profiles [76].Various research groups have aimed to evaluate the genetic and molecular profiles of EOC cell lines, and, on occasion, propose histotype reclassification [77–84].

Two of the most commonly studied EOC cell lines, SK-OV-3 and A2780, were originally classified as HGSOC; however, their mutational profiles were found to diverge significantly from those of other tumours classified as HGSOC [77, 80]. The absence of *TP53* mutation, and presence of mutations in *ARID1A* and mutations and amplifications in *PIK3CA*, which are more strongly associated with other histotypes such as OCCC and EnOC, suggested that these cell lines be re-classified [79]. While A2780 cells are now commonly classified as an EnOC cell line, SK-OV-3 cells continue to be referred to as HGSOC or used without reference to a histotype in many publications, including in over 50% of the EOC CRISPR-KO library screens presented in this review. The use of SK-OV-3 cells as a model of HGSOC without further validation in other *bona fide* HGSOC models therefore limits the impact of these studies [80, 85].

Profiling of ES-2, originally classified and commonly used to model OCCC, has shown an absence of HNF-1β (upregulated in OCCC and considered an OCCC histotype-specific genetic biomarker), and an absence of *ARID1A* and *PIK3CA* mutations and/or copy number variations which has posed a question mark over its classification [83]. It remains unclear as to which histotype ES-2 should be assigned due to its immunohistochemical profile resembling EnOC [77], *TP53* mutations being indicative of HGSOC [83], and *BRAF* mutations that align with LGSOC [85]. Despite this controversy, ES-2 continues to be used as a model of WT *ARID1A* and *PIK3CA* OCCC [35, 36].

Prior to 2024, JHOC-5 was considered a WT *ARID1A* OCCC model; however, DepMap 24Q4 Public dataset reported a frameshift mutation introducing a premature stop codon in *ARID1A*, therefore revoking its WT *ARID1A* status [86]. The evolving landscape of the molecular annotations of cell lines highlights the importance of researchers remaining vigilant when using these models and also when interpreting published literature.

## Selection methods used in EOC CRISPR-KO library screens

Tumorigenesis is defined by six ‘hallmarks of cancer’: sustaining proliferation signalling, evading growth suppressors, activating invasion and metastasis, enabling replicative immortality, inducing angiogenesis, and resisting cell death [87, 88]. Following transduction and primary selection for CRISPR-KO transduced cells, selection pressures designed to segregate cells into populations capable of yielding information on the genes contributing to these hallmarks of cancer are applied. The following sections discuss methods of evaluating cancer cell proliferation, protein expression and metastasis mechanisms, as well as interrogating drug effects within the context of current EOC CRISPR-KO libraries.

### Evaluating genes involved in cancer cell proliferation

Evaluating essential cell survival genes is the simplest application of CRISPR-KO library screens. Following library transduction and selection, CRISPR-KO cells remain in culture for a defined period of time; often 8–15 cell doublings, with samples collected throughout the screen to evaluate enrichments or depletions in sgRNA representation [3, 34–36, 56]. This screening format is particularly useful for comparing genetic dependencies between different genetic backgrounds, such as different cell lines or CRISPR engineered single gene KOs. Simulated data from CRISPulator has suggested that the duration of selection in a proliferation-based screen is dependent on the desired screening directionality [59]. Genetic bottlenecks increase with passage number or repeated application of selection pressure. Consequently, shorter time-frames (less than 10 passages) and single applications of selection pressure should be used for drop-out screens to prevent over selection and the loss of significance of rare “hits”. On the contrary, enrichment screens demonstrate reliable detection of positively selected phenotypes in longer screens (more than 10 passages).

In a study aiming to investigate genetic dependencies of ARID1A KO in the OCCC cell line, RMG-1, cells were transduced with the TKOv3 library and maintained in culture for 14-days post-puromycin selection (approximately 8–10 doublings), optimising the screen for identification of negatively selected phenotypes [38]. Comparison of sgRNA representation between WT and ARID1A KO RMG-1 cells revealed that the depletion of sgRNAs targeting the E3 ubiquitin ligase adapter, *KEAP1*, reduced cell growth in an *ARID1A* mutant dependent fashion. Consequently, developing clinically viable KEAP1 inhibitors, could be a promising therapeutic avenue for *ARID1A*-mutant OCCC [5, 38].

The use of in vivo models such as in the first EOC CRISPR-KO library screen whereby GeCKOv2 transduced SK-OV-3 cells propagated for 7 days in vitro were intraperitoneally injected into nude mice at a coverage of 100x and tumours larger than 10 mm diameter extricated from the peritoneal cavity after 7 weeks, allows for a more complex translational screening approach [41]. This screen revealed a depletion of sgRNAs targeting karyopherin β-1 *(KPNβ1*), involved in the transport of cell-cycle mediators through Ran-GTPase. Downstream analysis where KPNβ1 was knocked down (KD) or inhibited resulted in decreased cell proliferation and reduced survival of SK-OV-3 cells, results of which were validated in CaOV-3 and OVCAR-3 HGSOC lines.

### Evaluating genes involved in metastasis

With the absence of accurate early detection methods for EOC, diagnosis is often at a late stage following metastasis to the extra-pelvic peritoneum and/or retroperitoneal lymph nodes (stage III, 84% of HGSOCs) and more distantly to extra-abdominal organs (stage IV, 12–21% of all ovarian cancer) [89]. Building an understanding of the genetic and molecular events involved in metastasis is therefore a key area of research that is potentially critical for improving patient prognosis. The mechanisms by which metastasis occurs through epithelial-mesenchymal transition (EMT), intravasation, extravasation, and colonisation are discussed in a review by Fares et al. [90] with ovarian cancer specific genetic and metabolic profiles discussed by Yeung et al. [91].

Importantly, advanced metastatic ovarian cancer is often associated with ascites, an accumulation of fluid in the peritoneal cavity [92]. Disseminated cancer cells can be found in ascites both as single cells and as spheroids as a consequence of anoikis resistance allowing them to survive outside of the primary tumour site [93]. Anoikis resistance, defined as the ability of a cell to resist detachment-induced death, has been a recent focus in HGSOC research and has been shown to positively correlate with tumour aggressiveness [94], highlighting that mutations involved in this resistance could be predictive of a poorer prognosis [95].

#### In vitro adhesion vs. suspension culture methods

The ability of cells to grow in suspension demonstrates their ability to grow independently of anchorage [96]. Suspension culture therefore mimics the tumorigenic property allowing cancer cells to detach from the primary site and survive in ascitic fluid, enabling migration to secondary locations, ultimately defining the ability of cells to evade anoikis. In the context of a CRISPR-KO library screening study, sgRNA representation is compared between cell populations grown under standard adhesion conditions or forced suspension conditions on laboratory tissue culture plasticware with ultra-low adhesion (ULA) coatings. In EOC models, the culture of PEO1 and SK-OV-3 cells for 5–10 days post-puromycin selection on poly-HEMA coated or commercial ULA plates, respectively, has identified that the KO of factors involved in fatty acid biosynthesis (ECHDC2 and ACADVL; [31]) allows cells to be maintained in suspension, and the loss of VSTM2L [42] and PCMT1 [48] prevents growth in suspension.

#### In vitro transwell migration assays

Transwell migration assays measure the ability of a cell to migrate from an upper chamber to a lower chamber through a semipermeable membrane or an extracellular matrix, usually in response to a defined chemoattractant, or to separate non-adherent cells from adherent populations [97]. SK-OV-3 CRISPR-Cas9 KO cells maintained in culture after puromycin selection were segregated into more (lower-chamber) or less (upper-chamber) migratory populations over 24 h [45]. A depletion of sgRNAs associated with transcriptional dysregulation and EMT pathways was shown in the non-migratory population, with Fc gamma receptor 1a (*FCGR1A*) showing strong significance [45]. Upon single gene KD, the loss of FCGR1A resulted in reduced expression of known metastasis proteins, N-cadherin, and vimentin [98–100], which suggested that FCGR1A functions in the translational regulation of migratory proteins in ovarian cancer.

#### In vivo methods to investigate genes related to metastasis

Like the in vivo study used to identify genes involved in tumour proliferation, research groups have used an in vivo CRISPR-KO library approach to compare sgRNA populations between primary and metastatic tumours. In brief, 1 × 10^6^ in vitro transduced SK-OV-3-GeCKOv2 cells were orthotopically injected into the left ovary of NOD-SCID mice and both primary and metastatic tumours from within the peritoneal cavity were dissected 40 days post injection [44, 46, 47]. The dissected cells were then expanded in vitro for second and third rounds of injection to identify genes involved in properties of sustained metastasis. Highly metastasised tumours showed a depletion of sgRNAs targeting *IL20RA*, *ITK*, and *HTR1E*, therefore suggesting a tumour suppressive role of the genes encoding these proteins in EOC.

At present, investigations of EOC metastasis and anoikis resistance pathways using CRISPR-KO library screening techniques have been predominantly conducted using SK-OV-3 cells, not in small part because of their demonstrated ability to form human tumour xenografts in mouse models [101]. The controversy, as previously discussed, surrounding the use of SK-OV-3 for EOC research, limits the translational application of these results and leaves a gap in the field regarding this research methodology whereby studies of metastasis and anoikis resistance remain to be conducted in robust EOC cell line models of a clearly defined histotype.

### Interrogating drug effects

Drug resistance is one of the leading contributors to poor prognosis of EOC, with most patients developing recurrent disease after initial cytoreductive surgery and chemotherapy with platinum- and taxol-based drugs [102]. Consequently, a primary focus of ovarian cancer research has been understanding the mechanisms involved in both resistance and sensitivity to common therapeutics [103–105].

**Fig. 2:**
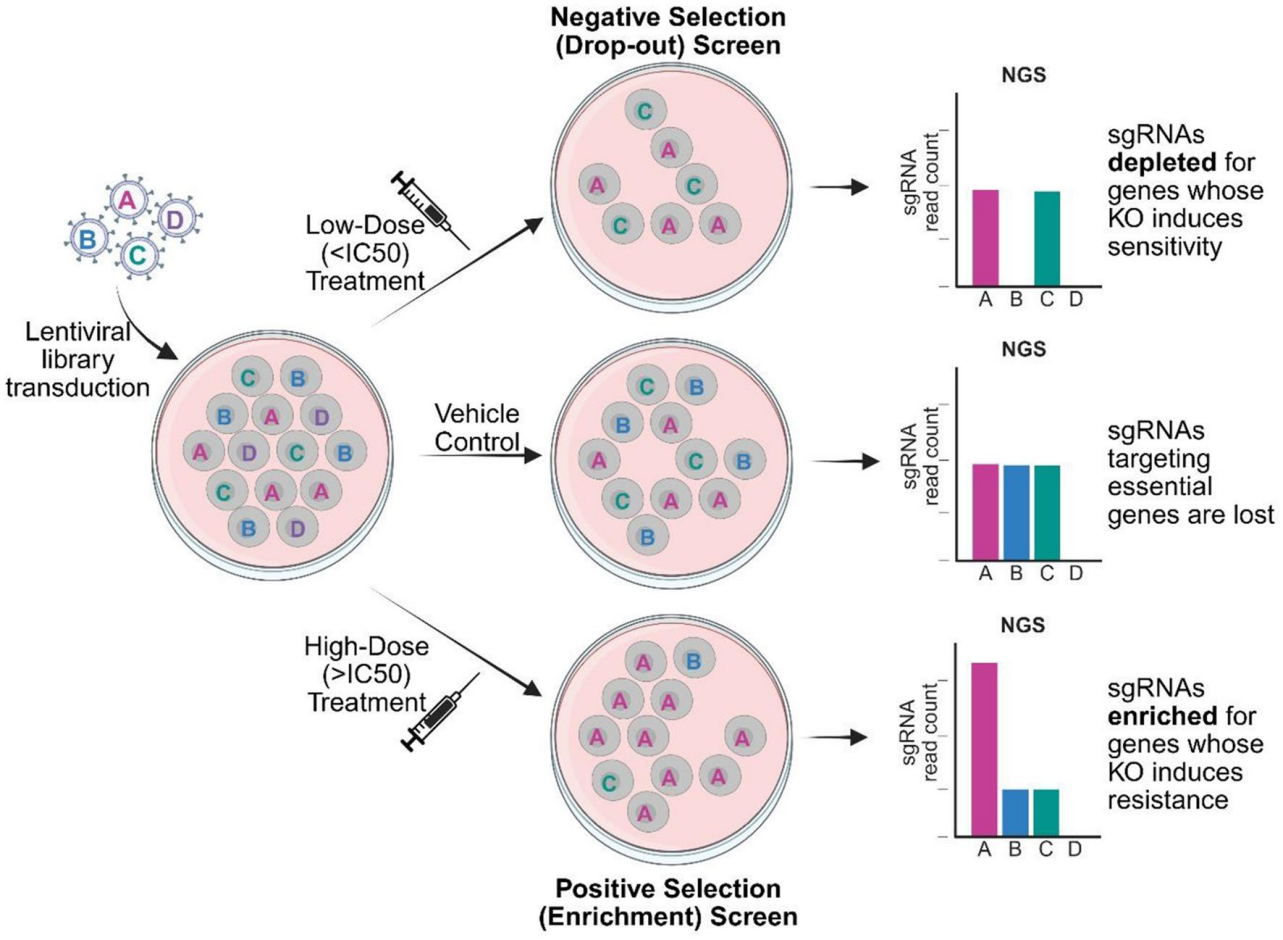
Schematic representation of CRISPR-knockout library screens to interrogate drug effects. Cells are first transduced with a lentiviral library containing sgRNAs targeted to different genes (A, B, C, and D depicted in single cells for illustrative purposes). sgRNAs targeting essential genes will be lost from all treatment groups (depicted by loss of essential “gene D” KO (knockout) cells). sgRNAs which are depleted in the low-dose treatment group in comparison with the control reflect genes whose KO induces sensitivity (depicted by loss of “gene B” KO cells). sgRNAs which are enriched in the high-dose treatment group in comparison to the control (depicted by increases of “gene A” KO cells) reflect genes whose KO induces resistance. Abbreviations: IC (inhibitory concentration), NGS (Next Generation Sequencing), sgRNA (single guide RNA)

CRISPR-KO library screens can be designed to identify genes involved in drug resistance and sensitivity by treating library transduced cells with defined drug concentrations and comparing sgRNA representations between vehicle control and treatment groups [19]. Due to the large-scale nature of CRISPR-KO library screens, it is usually not feasible to evaluate a range of different drug concentrations in a single study. Consequently, a single concentration is usually used, with the dose and drug mechanism dictating the dynamic range and duration of the screen. Low doses are used for negative selection screens and focus on identifying genes whose KO induces sensitivity whereas high doses are used for positive selection screens and reveal genes whose KO induces resistance (Fig. 2).

#### Drop-out screens using low dose drug treatment

Using a low concentration drug treatment that results in 5–50% cell death [18, 19], drop-out screens focus on the depletion of sgRNAs between treatment and control groups. Here, the loss of sgRNAs following drug treatment highlights GOIs that, when knocked out, increase the sensitivity of a cell to treatment (Fig. 2). This reveals potential pathways of inhibition that can be exploited in the development of new cancer therapeutics. In the context of EOC research to date, drug exposure has taken one of two formats. (1) Brief exposure (e.g. 72 h) followed by a period of recovery (e.g. 10 cell-doublings), enabling identification of genes essential for survival at first exposure [27]. (2) Prolonged exposure for a period of 7–10 days with cells treated every 3–4 days, providing time for genes essential to survive repeat exposures to be revealed [29, 33, 37, 39].

CRISPR-KO library screens using these methods have identified synergistic therapeutic targets with cisplatin, carboplatin, and paclitaxel in HGSOC [27, 29], investigated resistance mechanisms of the MEK inhibitor, trametinib in LGSOC [33], and explored targets to induce a ‘BRCAness’ phenotype in BRCA1 sufficient EOC cell lines to render cells more susceptible to PARP inhibition [39]. Moreover, sgRNA dropout drug treatment screens can be beneficial for identifying disease biomarkers for increased sensitivity to established and novel treatment regimens for improving personalised medicine approaches. In 2019, ceralasertib, an ataxia telangiectasia and rad3-related (ATR) protein inhibitor entered a phase II clinical trial for patients with recurrent gynaecological cancer with *ARID1A* mutation (NCT04065269; [106]). Using the OCCC cell line TOV-21G, researchers discovered that KO of protein phosphatase 2A (PP2A) subunits sensitised cells to ceralasertib, subsequently proposing that patients be screened for *PP2A* subunit mutations in addition to *ARID1A* mutations to increase the likelihood of ceralasertib treatment success [37].

#### Enrichment screens using high dose drug treatment

Using higher, but still sublethal, drug concentrations, resulting in > 50% cell death, and often as high as 80%, allows for a CRISPR-KO library screen to focus on detecting guides enriched in treatment groups, resulting in the identification of genes whose KO increases drug resistance (Fig. 2) [18, 19]. In EOC research, published screening formats resemble that of drop-out screens with the key difference being drug dose [32, 33, 40, 43]. Due to the higher doses used, positively selected cells may need to be grown for a longer period post-initial exposure to clearly identify enrichments and maintain high enough coverage for sequencing. This can be challenging if the drug mechanism works through cell cycle inhibition.

Such studies can be useful in identifying genes that may contain loss-of-function mutations in patient populations that can predict a negative response to treatment. The zinc finger protein 87 (ZNF587B), and the cell surface localised sulfatase 1 (SULF1) in A2780 and SK-OV-3 cell lines, respectively [40]; perturbations to NOTCH signalling in LGSOC [33]; and the transcriptional regulation of DYNLL1 by ATMIN in *BRCA1*-mutant EOC [32] have all been identified using this approach. Furthermore, positive selection enrichment screens are used to discern the mechanisms of action of proposed drug treatments. EOC studies have utilised this technique to provide evidence for repurposing of the antibiotic elaiophylin for the treatment of ovarian cancer by demonstrating that it exerts its cancer cell inhibitory effects through SHP2 binding and resultant inhibition of the MAPK signalling pathway [43].

### Evaluating genes involved in regulators of membrane protein expression

Fluorescence-activated cell sorting (FACS) of post-library transduced cells into populations of reduced and enhanced expression of known membrane proteins of interest is an alternative read-out of CRISPR-KO library screens [19, 107]. Here, differentially represented sgRNAs, both enriched or depleted, in either population can reveal regulators of membrane protein expression, identifying potential synergistic targets to increase efficacy of targeting these proteins.

EOC-based CRISPR-KO library screens have used this approach to evaluate regulators of expression of cell surface immune receptors by sorting stained CRISPR-KO library selected cells into populations of low and high expression [28, 49]. Regulators of CD276 expression in SK-OV-3 cells were evaluated by sorting cells 7-days post-puromycin selection at 4000x coverage into low and high CD276 populations and comparing sgRNA enrichment with the unsorted control sample [49]. Similarly, regulators of CD24 expression were evaluated by sorting and sequencing cells with low CD24 expression 15-days post-transduction at > 1300x coverage [28]. CRISPulator simulations have shown that the most important parameters for FACS-based CRISPR-KO library screen performance are bin size and adequate coverage at time of sorting, parameters which depend on experimental question, as well as time and budget [19, 59].

## Analysis of CRISPR-KO library screening data

Generating meaningful conclusions from NGS data created from a CRISPR-KO library screen using the aforementioned techniques requires extensive data analysis. Numerous bioinformatic pipelines exist and as a result, the following sections will focus specifically on those documented in current EOC CRISPR-KO library screen literature. For all pipelines, the quality of NGS data has a large impact on the statistical power and subsequent capacity for identifying true-positive GOIs [24]. NGS read-depth refers to the number of times each base is read during sequencing [108]. Consequently, much like maintaining sgRNA coverage during a CRISPR-KO library screen, adequate read-depth during NGS is required to detect low-frequency changes in sgRNA representation, minimise noise, and accurately quantify sgRNA counts. It is recommended that sequencing occurs at a read depth equal to the desired sgRNA coverage; however, where a strong positive selection pressure is used, resulting in high sgRNA loss, selected samples can be sequenced at a lower depth [24].

### Identifying GOIs from NGS data

NGS data is typically supplied as large FASTQ files of short sequencing reads representing sgRNA amplicons found within the cell population. To evaluate differential sgRNA representation between two or more populations, reads must be mapped to the reference sgRNA library and counted [19]. A number of validated pipelines to map and analyse NGS data fom a CRISPR-KO library screen exist, with algorithms commonly used for analysing EOC focussed CRISPR-KO library screening data outlined in Table 3. MAGeCK (Model-based Analysis of Genome-wide CRISPR-Cas9-Knockout) contains an inbuilt function for read mapping and counting allowing raw FASTQ files to be used as the data input. Other methods require FASTQ data to be processed prior to sgRNA ranking using common sequence alignment tools such as Bowtie or BWA (Burrows-Wheeler Aligner) [109, 110]. Each algorithm employs different statistical models for evaluating sgRNA representation between two or more datasets, with most evaluating both sgRNA enrichment and depletion [111].

**Table 3:** Common bioinformatic algorithms used for analysing EOC focussed CRISPR-KO screening data

Algorithm	Description	Language	Copy No. Correction	Data Input	Refs
RIGER	sgRNAs are ranked by a signal to noise-ratio and genes are ranked according to a Kolmogorov-Smirnov test	Java	No	FASTQ data processed into a tabulated read count file	[112]
MAGeCK	sgRNAs are ranked by a negative binomial distribution and genes are ranked according to robust rank aggregation (RRA).	Python, R	Yes	Raw FASTQ files	[113, 114]
BAGEL	sgRNAs are ranked by distribution to a reference gene set and genes are ranked using the Bayesian classifier	Python	No	FASTQ data processed into a tabulated read count file	[115]
STARS	Ranked sgRNAs are used to calculate gene scores using the probability mass function of a binomial distribution	Python	No	List of sgRNAs ranked based on essentiality	[51]

Abbreviations: Bayesian Analysis of Gene Essentiality (BAGEL), Model-based Analysis of Genome-wide CRISPR-Cas9-Knockout (MAGeCK), RNAi gene Enrichment Ranking (RIGER), STatistical Analysis of Reported hits from Screens (STARS)

Some more recent algorithms such as CERES, Chronos, and MAGeCKFlute use publicly available, or user-supplied, copy number data to enable copy number alterations to be considered within the analysis pipeline as these can affect the number of CRISPR-Cas9-induced DNA cuts, which can have implications on cellular response independent of genomic loci, increasing the risk of false-positives [114, 116–118]. This can be particularly important when EOC models are compared, as copy number alterations are known to present in a histotype-specific manner and are especially characteristic of HGSOC [119].

Owing to different statistical analysis methods, different algorithms are likely to report differing levels of significance. Consequently, understanding the analytical framework of an algorithm is critical in impactful reporting of CRISPR-KO library screen data. Further, increased confidence in results could be obtained through evaluating the overlap of GOIs between multiple methods [111]. Precise methodology, applications, and systematic comparisons of these algorithms, along with others not yet used to analyse EOC based CRISPR-KO library datasets, have previously been published within the literature [68, 111, 120, 121].

### Prioritisation of GOIs

CRISPR-KO library screens may yield a large number of GOIs which require careful curation in order to select those most interesting to the researcher for subsequent validation and characterisation. To date, researchers have used a range of methods to filter CRISPR-KO library data down to a discrete list of novel GOIs for further biological validation.

#### Gene set enrichment analysis

Gene set enrichment analysis (GSEA) enables the identification of biological functions and molecular pathways correlated with differentially expressed genes (DEGs) identified from the analysis of CRISPR screen data [122]. Several GSEA methods exist, with the majority of EOC CRISPR-KO library studies utilising the gene ontology (GO) [123, 124] and Kyoto Encyclopedia of Genes and Genomes (KEGG) databases [125, 126]. GO enrichment analysis can be used to evaluate clusters of DEGs categorised into ‘biological process,’ ‘molecular function,’ and ‘cellular component’ [124] through the PANTHER (protein analysis through evolutionary relationships) classification system [127]. KEGG pathways describe the molecular networks associated with GOIs, and KEGG Mapper can be used to interpret this information from CRISPR-KO library screening data [128]. With the evolving nature of gene ontology, enrichments may change over time; therefore, it is recommended that caution be taken in regard to interpretation [129]. Alternative methods such as SAFE (Significance Analysis of Function and Expression), sigPathway, CAMERA (Correlation Adjusted Mean Rank) and FANGS (Flexible Algorithm for Novel Gene set Simulation) have been systematically compared [122].

#### Comparison with publicly available CRISPR datasets: DepMap

The Broad Institute’s Cancer Dependency Map project (DepMap) is a high throughput CRISPR-KO library screening project aimed at identifying cancer dependencies to prioritise therapeutic targets. Initially established following RNA inhibition (RNAi) library annotation of 501 cancer cell lines [130], DepMap has expanded to include over 1900 cancer cell lines, encompassing 75 ovarian cancer cell lines annotated with CRISPR-KO library screening data [86]. This CRISPR-KO library data was generated using Avana4 and Human Improved Genome-Wide Knockout CRISPR Library v1. While the analysis of DepMap data in ovarian cancer cell lines can be used as a ‘stand-alone’ data source for providing a research foundation [131–135], many groups use DepMap data to cross-reference results between ovarian cancer and as a pan-cancer analysis tool to decipher the specificity of an ovarian cancer dependency, potentially strengthening the association of a genetic hit as a cancer property [35, 36, 38].

#### Insights from primary tissue and clinical data

The Human Protein Atlas [136, 137] and Gene Expression Omnibus (GEO) [138] allow researchers to decipher the relevance of ovarian cancer GOIs. This stage of selection has led to the exclusion of a statistically significant false-positive hit, *POTEC*, shown to only be expressed in testis, which was identified in A2780 and SK-OV-3 GeCKOv2 transduced cells treated with cisplatin, from being further studied for its relevance in cisplatin resistant ovarian cancer [40].

Furthermore, many studies have investigated the tumour-specific expression of their top statistically significant DEGs to evaluate the potential mutational burden of these genes on tumorigenesis and overall patient survival. Interactive online portals such as the National Cancer Institute Genomic Data Commons (GDC), cBioPortal, and Kaplan-Meier Plotter are repositories of publicly available clinical, genomic and transcriptomic data which can be combined with library screening results in order to prioritise downstream investigations [139–146]. At present, these repositories generally have limited data on EOC, with the exception of HGSOC being the most frequent histotype.

Using publically available datasets researchers evaluated clinical correlations of GOIs from CRISPR-KO library screens in EOC cell lines and reported that increased expression of TIGAR, VSTM2L, and PCMT1, and the reduced expression of HTR1E, ITK, and DYNLL1 (specifically in patients with *BRCA1*-mutations), were correlated with poor prognosis [32, 39, 42, 46–48]. Combing the afformentioned bioinformatic techniques to analyse CRISPR-KO library screening data can streamline downstream laboratory research, reduce costs, and increase the likelihood of clinically relevant discoveries.

## Translational potential of CRISPR-KO library screening for EOC

To date, the results from published EOC CRISPR-KO library screens have defined starting points for further research into novel disease biomarkers predictive of disease state and response to treatment, as well as proposing promising new therapeutic avenues. Functionally validating CRISPR-KO library screen “hits” using in vitro and/or in vivo methods following their identification is critical for confirming KO association with the observed phenotype. This is often done through single gene RNAi KD or CRISPR-Cas9 KO experiments using the same or different guide sequences included in the screen, and using a functional read-out reflective of the original screening format. These assays can include proliferation, migration, invasion, clonogenicity, and drug response, and will depend on the function of the GOIs, as well as the primary experimental question. In the case of all, it is important to show that KD or KO results in a significant decrease in “hit” expression at both the RNA (e.g. qRT- PCR) and protein (e.g. western blot) levels.

This section discusses some of the key translational applications of results from EOC CRISPR-KO library screens, highlighting where some of these functional validation assays have been used. At present, translational applications where the focus has been on biomarker identification, and some evaluating drug response mechanisms, have been conducted in incorrectly characterised cell line models [39, 40, 42–49]. Consequently, details of these studies have been omitted from this section in favour of presenting data more representative of defined EOC histotypes.

### Integration in multi-omics pipelines

Integrating CRISPR-KO library screens into multi-omics pipelines provides a powerful means of linking gene perturbations (genomics) with diverse molecular phenotypes across transcriptomics, proteomics, epigenomics, metabolomics, and microbiomics [15, 147]. Since cancer is a multifaceted disease, multi-omics approaches allow an understanding of pathways contributing to disease rather than focussing on single genetic perturbations, potentially increasing the clinical relevance of findings.

#### Factors affecting resistance to Anoikis

Bioinformatic analysis of a CRISPR-KO library screen in the HGSOC cell line, PEO1, cultured under conditions favouring adhesion or suspension, revealed a depletion of sgRNAs targeting genes associated with fatty-acid metabolism (*ACADVL* and *ECHDC2*) in suspension compared with adhesion cultures [31]. Primary validation of these GOIs was conducted using small hairpin RNA (shRNA) KD in the cell line PEO1, and a second HGSOC cell line, OVCAR-4, and showed reduced proliferation of suspension cells. Concordance was shown between CRISPR-KO library screen results, global non-targeted metabolomic mass-spectrometry, and RNA-seq evaluating transcriptomic differences between adhesion and suspension cell cultures, with clinical relevance of the library “hits” shown through comparison with publicly available ovarian cancer microarray and expression data. Along with identifying novel connections with fatty-acid metabolism, this approach was able to show that the specific upregulation of a previously proposed biomarker of HGSOC, cellular retinoic acid binding protein 2 (CRABP2), is linked with cancer cell dissemination [31, 148]. This has demonstrated the ability of CRISPR-KO library screens to predict biomarkers of disease state when the KO of a key factor leads to enhanced or inhibited tumorigenic properties; however, additional compelling research focussing on biomarker identification using this technique is yet to be reported.

#### Anti-cancer properties of repurposed drugs

The integration of CRISPR-KO library screens into multi-omics pipelines is also beneficial for understanding anti-cancer properties of drugs. One study used a CRISPR-KO library screen aimed at investigating biomarkers of HGSOC cell viability in response to the antimalarial agent, hydroxychloroquine (HCQ) to develop a more comprehensive understanding of patient populations most likely to benefit from its use [30]. HCQ has gained traction in its repurposing as a pan-cancer therapeutic with clinical trials in combination with standard regimens dating back to 2008; however, little is known about mechanisms behind its potential as a cancer therapeutic [149]. Mechanisms of developed HCQ resistance were examined using deep exome sequencing, bulk mRNA sequencing, and single-cell RNA sequencing following repeated exposure to HCQ, with results being cross-referenced to a CRISPR-KO library screen in the HGSOC cell line, OVCAR-3 to prioritise the most biologically relevant genes [30]. Aerobic glycolysis pathways and the exocytosis network were shown to be transcriptionally upregulated and CRISPR-KO library screens showed that the KO of genes relevant to these pathways results in HCQ sensitivity. Specifically, NEK2 KO was shown to correlate with increased sensitivity to HCQ and given that NEK2 overexpression in a range of cancers acts as a biomarker of poor prognosis, it was proposed that this could be extended as a predictive indicator of patient response to HCQ [150]. With early clinical trials of HCQ in combination with Itraconazole (a clinically approved antifungal medication) in platinum-resistant EOC patients showing no anti-tumour effects (NCT03081702; [151]), future selection of clinical trial participants could exclude those with NEK2 overexpression, along with other targets identified from this study.

### Promising novel therapeutic avenues

The ‘druggable genome’ includes 22% of the annotated protein-coding genes stratified into three tiers of ‘druggability’ [152]. These are genes encoding targets with approved therapeutic drugs and drugs in clinical trials; genes encoding targets with known small-molecule binding partners or high sequence identity to those with approved drugs; and genes encoding secreted and extracellular proteins or proteins that are members of known druggable gene families such as G-Protein Coupled Receptors, hormone receptors, ion channels, and kinases. Although targets that are currently not actionable due to lack of a suitable drug for clinical use are not discounted, prioritisation of investigations on the basis of druggability may accelerate clinical translation.

Actionable targets present promising opportunities for novel therapeutic avenues. Based on the current literature, three genes (*KEAP1*, *BCL2L1*, and *CSNK2A2*) falling into two categories described below, satisfy these requirements. At present, mechanistic insight into the targeting of these genes for the treatment of ovarian cancer remains limited; however, their identification in CRISPR-KO library screens highlights a need for further investigation.

#### Synthetic lethal opportunities for treating *ARID1A* mutated OCCC

**Fig. 3:**
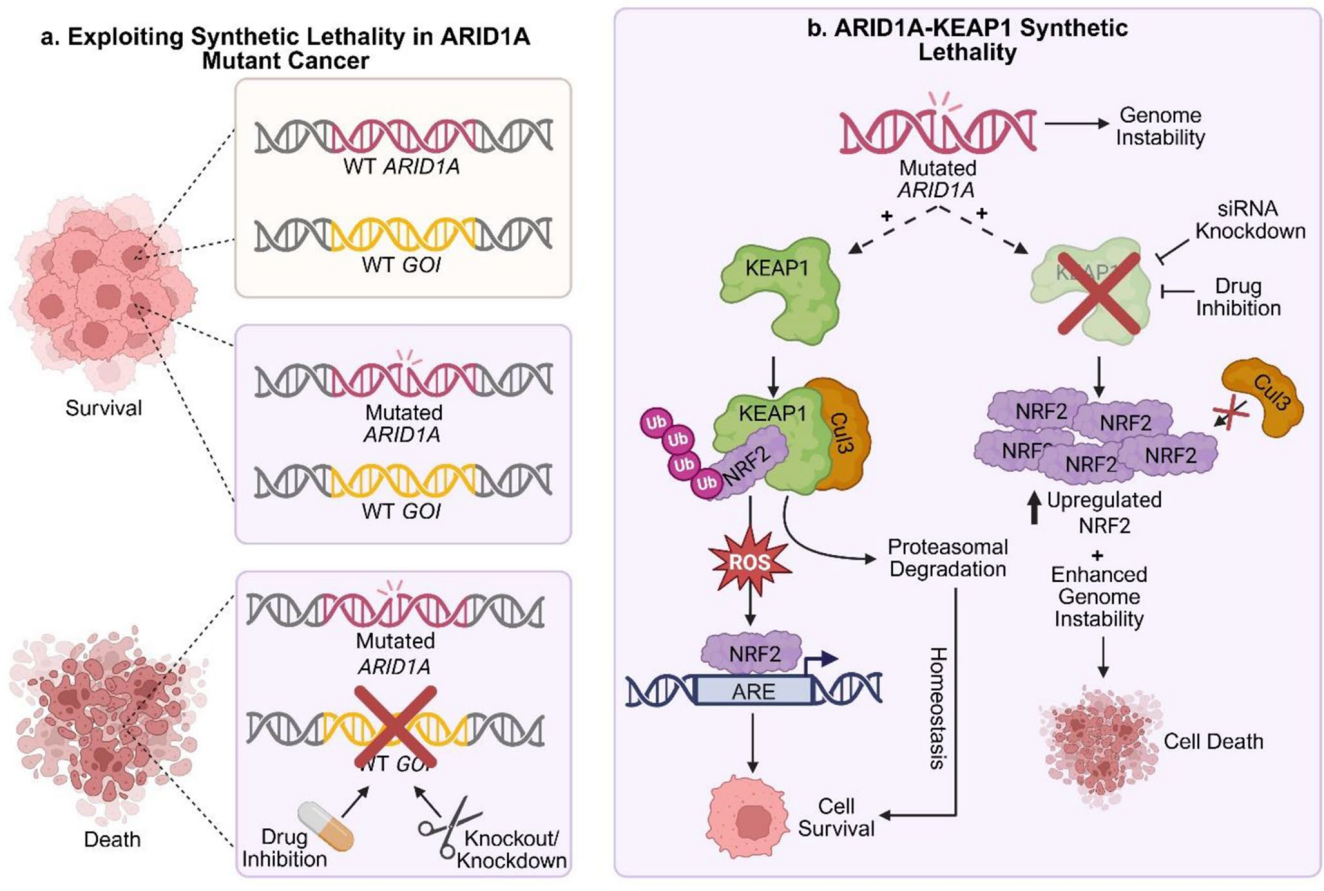
Example of a proposed mechanism of synthetic lethality in ARID1A mutant tumours. (**a**) Exploiting synthetic lethality in ARID1A mutant cancer. Synthetic lethality is caused by the dual inactivation of genes from a synthetic lethal pairing. Cell survival is achieved where neither, or only one of the genes within a synthetic lethal pair are perturbed. An ARID1A (pink) synthetic lethal gene of interest (GOI; yellow) can be identified through directed gene knockout or knockdown, or drug inhibition in the presence of mutant ARID1A resulting in cell death. (**b**) ARID1A-KEAP1 synthetic lethality - proposed mechanism of action. Functional KEAP1 acts as a Cul3 ligase adaptor to specifically target NRF2 for degradation. In the presence of reactive oxygen species (ROS), NRF2 is translocated to the nucleus to activate the transcription of genes under the control of the antioxidant response element (ARE). The balance of NRF2 degradation and ARE activation allows cell survival. When KEAP1 is perturbed (through knockdown or drug inhibition), polyubiquitination of NRF2 is inhibited resulting in increased NRF2. Enhanced genomic stability is observed resulting in cell death [38, 156]

Harnessing synthetic lethality is at the forefront of precision oncology [153]. An example of use of this strategy is in OCCC where ARID1A loss-of-function mutations are characteristic of disease (Fig. 3a) [154]. Fournier et al. [38] investigated synthetic lethal ARID1A targets using a TKOv3 library screen in an RMG-I CRISPR-Cas9 ARID1A KO isogenic cell line pair. Bioinformatic analysis confirmed known synthetic lethal partners DDX19A, SMARCC1, and FAAP24, and identified a novel ARID1A synthetic lethal partner, the Cullin 3-based ubiquitin E3 ligase adaptor, Kelch-Like ECH Associated Protein 1 (KEAP1) [38, 130, 155].

Confirming synthetic lethal relationships between genes can be challenging as their simultaneous KO is expected to result in cell death, preventing mechanistic insights into the cause of lethality from being investigated. Consequently, downstream analysis of synthetic lethal interactions often relies on gene KD and/or drug treatment with inhibitors known to interact with the protein encoded by a defined GOI. Both methods were used to validate KEAP1 as an ARID1A synthetic lethal partner showing that KEAP1 pertubations through KD and inhibition (using the “research use only” compound, AI-1) reduced proliferation and increased expression of NRF2 (nuclear factor erythroid 2-related factor 2) (Fig. 3B).

The KEAP1-NRF2 stress response pathway has been extensively documented [157], and both loss-of-function and gain-of-function mutations in KEAP1 are common in cancer, most frequently in lung squamous cell carcinoma [158, 159], with some studies also reporting *KEAP1* mutations in EOC [156, 160]. Despite this, activation of the NRF2 pathway through inducible expression of a NRF2 variant with reduced KEAP1 binding affinity did not show a significant ARID1A-KO dependent reduction in proliferation. Instead, it was proposed that ARID1A-KEAP1 synthetic lethality results from dual enhancement of genomic instability as increased DNA replication stress was shown only in ARID1A-KO cells treated with AI-1 (Fig. 3B) [38]. Results from this study have given merit for future development of clinically viable KEAP1 inhibitors which could be highly promising for treating ARID1A-mutant OCCC and advancing OCCC precision medicine approaches.

#### Dual targeting strategies for overcoming chemoresistance

Acquired chemoresistance occurs when cancer cells undergo various genetic and epigenetic modifications to avoid death from chemotherapeutic agents [161]. CRISPR-KO library screens can be used to identify genes contributing to acquired drug resistance and investigate ways of targeting these genes to sensitise cells to standard-of-care chemotherapy. sgRNAs targeting *BCL2L1* and other BCL-2 family proteins were shown to be significantly depleted in HGSOC (Kuramochi and OVSAHO) CRISPR-KO library cells treated with cisplatin and/or paclitaxel with the opposite being true in a parallel overexpression screen [29]. In overexpression models it was shown that the overexpression of both BCL2L1 and BCL2L2 individually correlated with a reduction in cisplatin and paclitaxel sensitivity, and a dose dependent reduction in caspase activity. This finding is consistent with many previous studies evaluating the anti-apoptotic nature of BCL2 proteins. Further downstream experiments looked at the effects of combining BCL-2 inhibitors in clinical development with traditional chemotherapies in cell viability assays which showed a demonstrated ability of this synergistic treatment to reduce cell viability. While no novel mechanisms were annotated in this study, connections with anti-apoptotic processes in HGSOC were strengthened.

OVCAR-8-Brunello KO cells treated with carboplatin concentration nearing IC50, exhibited depletion of sgRNAs targeting several kinases and receptors [27]. Of particular interest was *CSNK2A2* which encodes the alpha prime (α’) subunit of CK2, a serine-threonine kinase with a broad range of targets involved in various physiological processes [162]. Inhibition of CK2 through both CSNK2A2 KO and inhibition with silmitasertib (CX-4945), a CK2 inhibitor, in combination with carboplatin, reduced the viability of both OVCAR-8 and COV362 cells, both of which harbour mutations in retinoblastoma transcriptional corepressor 1 (RB1), a known tumour suppressor protein, mutated in approximately 10% of HGSOC [163]. This highlighted the ability of CK2 targeting to sensitise certain HGSOC cells to carboplatin [27]. They further demonstrated that CK2 inhibitors also exerted positive synergy with multiple PARP inhibitors in the same cell lines, with the strongest sensitising effect shown with saruparib. The CK2 inhibitor silmitasertib has been identified as a potential sensitiser for dasatinib, cisplatin, and carboplatin in pre-clinical EOC cell lines [164]. With silmitasertib currently undergoing clinical trials for various malignancies, including basal cell carcinoma (NCT03897036), cholangiocarcinoma (NCT02128282), medulloblastoma (NCT03904862), and kidney cancer (NCT03571438), future trials could consider its use in RB1-deficient HGSOC.

## Summary

Human whole-genome CRISPR-KO library screening is an emerging technique in the field of ovarian cancer which provides a platform for building a genome-level understanding of disease, aiding in target-discovery for precision medicine approaches. This is key in improving therapeutic options for treating EOC due to high genetic diversity between histotypes and their high propensity for acquired chemoresistance [4, 102]. CRISPR-KO library screens have the potential to generate an abundance of data and at present have contributed to multi-omics pipelines, revealed novel ARID1A synthetic lethal partners in OCCC, proposed biomarkers for improving treatment selection criteria, and identified molecular targets likely to be synergistic with standard-of-care chemotherapies [27, 29, 30, 38].

Obtaining impactful results in preclinical ovarian cancer research requires the use of models which accurately reflect the disease in question because of the high genetic diversity. Many of the current CRISPR-KO library screens focussing on EOC lack clear distinction of histological subtype which limits the translational potential of these findings. Consequently, future studies adopting this methodology should prioritise model selection to allow genetic dependencies to be mapped to specific EOC histotypes. As these are published, cross-study meta-analyses could be highly beneficial in identifying further GOIs and uncovering shared or subtype-specific vulnerabilities, even from studies which initially lacked histotype distinction [165].

Despite their value in unbiased, early target identification, CRISPR-KO library screens targeted at the genome wide level are costly and time consuming due to the number of cells required to maintain adequate sgRNA coverage throughout the screen [19]. Focussed library screens with a pre-defined question are directed towards a smaller subset of genes and can offer a more targeted approach for certain scientific objectives, reducing the magnitude of the screen [23]. This more targeted approach has been used in a limited number of EOC cell lines including for the interrogation of DNA damage repair pathway genes in a PEO1, BRCA2 restored isogenic pair [166]. Many of the downstream selection methods discussed in this review can be applied to these more focused libraries. Furthermore, focussed library screens can be used following a whole-genome screen upon hypothesis establishment as a method of validating results [54]. To our knowledge, this method of validation is yet to be used to study EOC.

## Conclusion and future directions

CRISPR-KO library screens are poised to significantly advance our understanding of EOC, as well as identify new therapeutic opportunities. Through careful model selection, optimisation of screening parameters, as well as strategic downstream validation, these tools can drive more effective, histotype-specific therapeutic interventions for improving patient outcomes. This review has discussed key methodological considerations for utilising whole-genome CRISPR-KO library screens in early target discovery, drawing on techniques from EOC-focused studies, with broader applicability to other cancers where effective treatment options remain limited.

To date, most histotype-directed CRISPR library screens have focussed on HGSOC and OCCC, leaving less frequently studied subtypes including EnOC, LGSOC, and MOC underexplored. Additionally, since EOCs often progress through acquired chemoresistance, the use of drug-resistant cell lines may reveal resistance mechanisms and synthetic lethal strategies for overcoming resistance. As the genomic landscape of EOC continues to be unravelled and molecular drivers are identified, CRISPR-KO library screens could be carried out in isogenic panels engineered with genetic driver mutations to disentangle gene-function relationships in a controlled genetic background. Lastly, focussing research on applying CRISPR screening technologies in primary-patient-derived cells and 3D models will be key in enhancing the clinical relevance of future findings. Applying these strategies could be advantageous in accelerating the development of precision therapeutics.

## Supplementary Information

Below is the link to the electronic supplementary material.

Supplementary Material 1

## Data Availability

No datasets were generated or analysed during the current study.
