# Supplementary material for: Revealing genetic drivers of ovarian cancer and chemoresistance: insights from whole-genome CRISPR-knockout library screens
Source: Cell Oncol (Dordr). 2025 Aug 28;48(5):1245–65. doi: 10.1007/s13402-025-01102-4 (PMC12528352; doi:10.1007/s13402-025-01102-4)
Supplement: Supplementary file 1 — Supplementary Material 1 [file 13402_2025_1102_MOESM1_ESM.docx]

**Revealing genetic drivers of ovarian cancer and chemoresistance: insights from whole-genome CRISPR-knockout library screens**Cellular Oncology

Tali S Skipper, Kristie-Ann Dickson, Christopher E Denes, Matthew A Waller, Tian Y Du, G Gregory Neely, Nikola A Bowden, Alen Faiz, Deborah J Marsh

**Corresponding author**

Deborah Marsh

Translational Oncology Group, School of Life Sciences, Faculty of Science, University of Technology Sydney, Ultimo, NSW, Australia

[deborah.marsh@uts.edu.au](mailto:deborah.marsh@uts.edu.au)

| Online Resource 1 Mutational profiles of ovarian cancer cell lines used to date in whole-genome CRISPR-knockout library screens | | | | | | |
| --- | --- | --- | --- | --- | --- | --- |
| Ovarian Cancer Histotype | Cell Line | Origin | Mutations in common tumour suppressor genes | Mutations in common oncogenes | Tumorigenic copy number amplifications | Tumorigenic copy number deletions |
| HGSOC | COV362 [1] | Metastatic pleural effusion | *APC*^2,4^  *ARID1B*^5^  *ATM*^4^  *BRCA1*^1,2,4,5^  *TP53*^1,2,4,5^ | *EGFR*^2^ | *ERBB2*^5^  *MYC*^1,2,5^  *PTEN*^5^ | *RB1*^1,5^ |
| HGSOC | JHOS-2 [2] | Metastatic lymph node | *FAT3*^4^  *BRCA1*^1,4,5^  *TP53*^1,4,5^  *NF1*^4,5^ |  | *MYC*^3,5^  *PIK3CA*^5^  *ZNF217*^5^ | *AKT2*^5^  *CDKN2A*^5^ |
| HGSOC | Kuramochi [3] | Metastasised ascitic tumour | *ATM*^2^  *SMAD4*^3^  *TP53*^1,3,4,5^  *BRCA2*^1,3,4,5^ | *GNAS*^4^  *LRRK2*^4^ | *BRAF*^5^  *ZNF217*^5^  *FGFR1*^3^  *KRAS*^1,3,5^  *MYC*^1,5^ | *BRCA1*^5^  *NF1*^5^ |
| HGSOC | OVCAR-3 [4] | Ascites | *PIK3R1*^3,5^  *STAG2*^3^  *TP53*^2,3,5^ |  | *AKT2*^3,5^  *CCNE1*^2,5^  *FGFR1*^3^  *KMT2B*^5^  *KRAS*^5^  *ZNF217*^5^ | *ARID1A*^5^  *ARID1B*^5^  *BRCA2*^5^  *CDK12*^5^  *ERBB2*^5^  *LRP1B*^3^  *MACROD2*^3^  *RB1*^5^  *SMARCA4*^5^  *TP53*^5^ |
| HGSOC | OVCAR-8 [5] | Unknown | *ATM*^4^  *FAT3*^4^  *APC*^4^  CREBBP^3^  *KRAS*^1,4,5^  *TP53*^1,3,4,5^ | *ERBB2*^1,3,4,5^  *CTNNB1*^1,4,5^  *KMT2B*^4,5^ | *MYC*^5^ | *MET*^5^  *WWOX*^3^ |
| HGSOC | OVSAHO [6] | Metastasised tumour | *ARID1A*^2^  *NF1*^4,5^  *TP53*^1,3,4,5^ |  | *BRAF*^5^  *FGFR4*^3^  *KMT2B*^5^  *MET*^5^  *PIK3R2*^5^  *SMARCA4*^5^  *ZNF217*^5^ | *BRCA2*^1,5^  *MACROD2*^3^  *RB1*^1,5^ |
| HGSOC | PEO1 [7] | Metastasised ascitic tumour | *CDK12*^5^  *NF1*^5^  *TP53*^2,5^ | *BRCA2*^2,5^  *KMT2B*^5^ |  |  |
| HGSOC | UWB1.289 [8] | Unknown | *TP53*^2,5^  *BRCA1*^2,5,6^  *NF1*^5^ |  |  |  |
| LGSOC | VOA-4627 [9] | Ascites | *PIK3CA*^6^  *TP53*^6^ | *KIT*^6^ |  |  |
| LGSOC | VOA-6406 [9] | Tumour of undocumented site | Mutational profile not documented | | | |
| OCCC | JHOC-5 [2] | Metastasised tumour from unknown site |  |  | *BRCA1*^5^  *MET*^5^ | *CDKN2A*^5^  *SMARCA4*^5^ |
| OCCC | OVISE [6] | Metastasised tumour from innominate bone | *APC*^4^  *ARID1B*^5^  *ARID1A*^1,3,4,5^  *PIK3CA*^3,4,5^ | *LRRK2*^4^  *NOTCH1*^3^  *PPP2R1A*^3,4^ | *BRCA1*^5^  *CCNE1*^5^  *MYC*^5^  *ZNF217*^5^ | *WWOX*^3^ |
| OCCC | RMG-I [10] | Ascites |  |  | *AKT2*^5^  *BRCA1*^5^  *CCNE1*^5^  *CDK12*^5^  *CDKN2A*^5^  *ERBB2*^1,5^  *KMT2B*^5^  *RAD51C*^3^  *RNF43*^3,5^ | *BRCA2*^5^  *CDC73*^3^  *CDKN2A*^3^  *EZH2*^3^  *LRP1B*^3^  *SMAD*^3^  *TP53*^1,5^  *WWOX*^3^ |
| OCCC | TOV-21G [11] | Tumour from the ovary | *PTEN*^1^  *FAT3*^4^  *ARID1B*^5^  *PIK3R1*^3^  *STK11*^3^  *RNF43*^4,5^  *KRAS*^1,2,4,5^  *ARID1A*^1,2,3,4,5^  *PTEN*^2,3,4,5^  *KMT2D*^4,5^  *NF1*^4,5^ | *ZNF217*^5^  *CCND1*^3^  *ERBB3*^3^  *FANCD2*^3^  *RNF43*^3^  *TPX2*^2^  *PIK3CA*^1,2,3,4,5^  *CTNNB1*^1,4,5^  *KMT2B*^4,5^ |  |  |
| EnOC | A2780* [12] | Ovarian endometrioid adenocarcinoma | *ARID1A*^2,3,4,5^  *ARID1B*^5^  *CSMD3*^4^  *FAT3*^4^  *KMT2D*^4,5^  *PTEN*^2,4,5^  SMARCA4^3,5^ | *BRAF*^4,5^  *BRCA2*^2^  *GNAS*^4^  *PIK3CA*^2,3,4,5^ |  | *FHIT*^3^  *WWOX*^3^ |
| HGSOC | SK-OV-3** [13] | Metastasised ascitic tumour | *ATM*^4^  *CSMD3*^4^  *APC*^4^  *FBXW7*^3,4^  *ARID1A*^1,3,4,5^  *TP53*^2,3,4,5^  *NF1*^2,4,5^ | *CTNNB1*^5^  *HRAS*^2^  *NOTCH2*^3^  *PIK3CA*^1,2,3,4,5^ | *CDK12*^5^  *ERBB2*^1,2,3,5^  *PIK3CA*^5^ | *BRAF*^5^  *CDKN2A*^3,5^  *CDKN2B*^3^  *MACROD2*^3^  *MET*^5^  *MLH1*^3^  *PTEN*^5^  *TP53*^5^ |
| OCCC | ES-2** | Tumour from the ovary | *APC*^2^  *KMT2D*^4,5^  *TP53*^1,2,3,4,5^ | *BRAF*^1,2,3,4,5^  *FANCD2*^3^  *MYC*^2^ | *CCND1*^3^ | *ESR1*^3^  *LRP1B*^3^  *WWOX*^3^ |
| Genes are listed in alphabetical order. Where available, the first documentation of the cell line is referenced in column 2.  *Reclassified histotype  **Original histotype in query  Chronological references: [14]^1^, [15]^2^, [16]^3^, [17]^4^, [18]^5^, Original publication classifying the cell line as referenced in column two^6^. | | | | | | |

**Online Resource References**

1. C.A. van den Berg-Bakker, A. Hagemeijer, E.M. Franken-Postma, V.T. Smit, P.J. Kuppen, H.H. van Ravenswaay Claasen, C.J. Cornelisse, P.I. Schrier, Establishment and characterization of 7 ovarian carcinoma cell lines and one granulosa tumor cell line: Growth features and cytogenetics. Int J Cancer 53, 613-620 (1993). doi: 10.1002/ijc.2910530415
2. K. Yamada, T. Tachibana, H. Hashimoto, K. Suzuki, S. Yanagida, H. Endoh, et al., Establishment and characterization of cell lines derived from serous adenocarcinoma (jhos-2) and clear cell adenocarcinoma (jhoc-5, jhoc-6) of human ovary. Hum Cell 12, 131-138 (1999)
3. T. Motoyama, [biological characterization including sensitivity to mitomycin c of cultured human ovarian cancers (author's transl)]. Nihon Sanka Fujinka Gakkai Zasshi 33, 1197-1204 (1981)
4. T. C. Hamilton, R.C. Young, W.M. McKoy, K.R. Grotzinger, J.A. Green, E.W. Chu, J. Whang-Peng, A.M. Rogan, W.R. Green, R.F. Ozols, Characterization of a human ovarian carcinoma cell line (nih:Ovcar-3) with androgen and estrogen receptors. Cancer Res 43, 5379-5389 (1983)
5. R.J. Schilder, L. Hall, A. Monks, L.M. Handel, A.J. Fornace, Jr., R.F. Ozols, A.T. Fojo, T.C. Hamilton, Metallothionein gene expression and resistance to cisplatin in human ovarian cancer. Int J Cancer 45, 416-422 (1990). doi: 10.1002/ijc.2910450306
6. T. Yanagibashi, I. Gorai, T. Nakazawa, E. Miyagi, F. Hirahara, H. Kitamura, H. Minaguchi, Complexity of expression of the intermediate filaments of six new human ovarian carcinoma cell lines: New expression of cytokeratin 20. Br J Cancer 76, 829-835 (1997). doi: 10.1038/bjc.1997.471
7. S.P. Langdon, S.S. Lawrie, F.G. Hay, M.M. Hawkes, A. McDonald, I.P. Hayward, et al., Characterization and properties of nine human ovarian adenocarcinoma cell lines. Cancer Res 48, 6166-6172 (1988).
8. C. DelloRusso, P.L. Welcsh, W. Wang, R.L. Garcia, M.C. King, E.M. Swisher, Functional characterization of a novel brca1-null ovarian cancer cell line in response to ionizing radiation. Mol Cancer Res 5, 35-45 (2007). doi: 10.1158/1541-7786.MCR-06-0234
9. A. Dawson, Targeted therapy in low-grade serous ovarian carcinoma:characterization of MEK inhibitor response in novel patient-derived cell lines. [Thesis] (2017). https://doi.org/10.14288/1.0319145.
10. S. Nozawa, K. Tsukazaki, M. Sakayori, C.H. Jeng, R. Iizuka, Establishment of a human ovarian clear cell carcinoma cell line (rmg-i) and its single cell cloning--with special reference to the stem cell of the tumor. Hum Cell 1, 426-435 (1988).
11. D.M. Provencher, H. Lounis, L. Champoux, M. Tetrault, E.N. Manderson, J.C. Wang, et al., Characterization of four novel epithelial ovarian cancer cell lines. In Vitro Cell Dev Biol Anim 36, 357-361 (2000). doi: 10.1290/1071-2690(2000)036<0357:COFNEO>2.0.CO;2
12. A. Eva, K.C. Robbins, P.R. Andersen, A. Srinivasan, S.R. Tronick, E.P. Reddy, et al., Cellular genes analogous to retroviral onc genes are transcribed in human tumour cells. Nature 295, 116-119 (1982). doi: 10.1038/295116a0
13. J. Fogh, G. Trempe, New Human Tumor Cell Lines, in: Fogh, J. (Eds.), Human Tumor Cells in Vitro, (Springer US, 1975) pp 115-159. https://doi.org/10.1007/978-1-4757-1647-4_5
14. S. Domcke, R. Sinha, D.A. Levine, C. Sander, N. Schultz, Evaluating cell lines as tumour models by comparison of genomic profiles. Nat Commun 4, 2126 (2013). doi: 10.1038/ncomms3126
15. C.M. Beaufort, J.C. Helmijr, A.M. Piskorz, M. Hoogstraat, K. Ruigrok-Ritstier, N. Besselink, et al., Ovarian cancer cell line panel (occp): Clinical importance of in vitro morphological subtypes. PLoS One 9, e103988 (2014). doi: 10.1371/journal.pone.0103988
16. E. Papp, D. Hallberg, G.E. Konecny, D.C. Bruhm, V. Adleff, M. Noe, et al., Integrated genomic, epigenomic, and expression analyses of ovarian cancer cell lines. Cell Rep 25, 2617-2633 (2018). doi: 10.1016/j.celrep.2018.10.096
17. B.M. Barnes, L. Nelson, A. Tighe, G.J. Burghel, I.H. Lin, S. Desai, et al., Distinct transcriptional programs stratify ovarian cancer cell lines into the five major histological subtypes. Genome Med 13, 140 (2021). doi: 10.1186/s13073-021-00952-5
18. A. McCabe, O. Zaheed, S.S. McDade, K. Dean, Investigating the suitability of in vitro cell lines as models for the major subtypes of epithelial ovarian cancer. Front Cell Dev Biol 11, 1104514 (2023). doi: 10.3389/fcell.2023.1104514
